# Preoperative mean platelet volume is a prognostic biomarker for survival in patients with gastric cancer: A cohort study

**DOI:** 10.1097/MD.0000000000030504

**Published:** 2022-09-09

**Authors:** Soomin An, Wankyu Eo, Ga Young Han, Sukyung Park, Sookyung Lee

**Affiliations:** a Department of Nursing, Dongyang University, Gyeongbuk, Republic of Korea; b College of Medicine, Kyung Hee University, Seoul, Republic of Korea; c Department of Music, Chang Shin University, Changwon, Republic of Korea; d Department of Nursing Science, Graduate School, Kyung Hee University, Seoul, Republic of Korea; e Department of Clinical Oncology, College of Korean Medicine, Kyung Hee University, Seoul, Republic of Korea

**Keywords:** gastrectomy, mean platelet volume, prognosis, stomach neoplasm

## Abstract

This study aimed to evaluate the prognostic potential of mean platelet volume (MPV) in gastric cancer (GC) patients. Patients with stage I–III GC who underwent gastrectomy were enrolled in this study. Cox regression analysis was performed to evaluate the determinants of overall survival (OS) and disease-free survival (DFS). The discriminative capacity of the model was determined using the Harrell concordance index (C-index). The net benefit of the model was validated using decision curve analysis (DCA). Data from 401 patients were analyzed. Multivariate Cox regression analysis revealed that age, stage, serum albumin level (ALB), perineural invasion (PNI) and MPV were determinants of both OS and DFS. The MPV model consisted of 5 covariates (age, stage, ALB, PNI, and MPV level), and the baseline model constituted the same covariates as the MPV model, except for the MPV level. C-indices for OS and DFS were higher in the MPV model than in the baseline model. When the models were validated using DCA, the MPV model showed a greater net benefit than the baseline model for nearly all the threshold probabilities. Age, stage, ALB, PNI, and MPV are prognostic factors for OS and DFS. The discriminative capacities for OS and DFS in the MPV model were higher than those in the baseline model, thus implying the clinical significance of the MPV level as a determinant of survival in GC.

## 1. Introduction

Gastrectomy is the primary treatment for gastric cancer (GC); however, it is associated with substantial relapse and mortality. As the specific biomarkers that can predict survival outcomes after gastrectomy remain unknown, establishing biomarkers that can accurately predict survival outcomes provides clinicians with valuable preoperative information that can help improve survival outcomes. Although the tumor-node-metastasis (TNM) staging system is regarded as the gold standard for predicting the prognosis of malignant tumors, it has disadvantages, such as different prognoses for the same tumor stage.^[1,2]^ Given the dissatisfaction with the biomarkers developed thus far and the incompleteness of the TNM system, further studies are required to establish simple but accurate novel biomarkers.

Platelets promote tumor growth and invasion.^[3]^ An increase in platelet count is a determinant of survival in patients with malignant tumors.^[4]^ With regard to platelet size, the mean platelet volume (MPV) has prognostic significance in specific tumor types.^[4–7]^ However, because of the heterogeneity of cancer types and cutoff values in studies, there is no clear consensus on the clinical value of MPV levels.^[5]^ In GC, although the pretreatment MPV level is not a prognostic determinant for overall survival (OS) based on multivariate analysis,^[8–11]^ the clinical value of the MPV level remains uncertain given the limited number of available studies, various cutoff values (from 9.85 to 11.65 fL), and various TNM stages. In addition, the prognostic role of MPV levels on disease-free survival (DFS) in stage I–III GCs has not been evaluated thus far.^[10]^ Thus, the clinical significance of MPV levels in patients with stage I–III GC remains unclear.

Therefore, this study aimed to evaluate the OS and DFS according to MPV levels in patients with stage I–III GC. Unlike previous studies, MPV levels were treated as continuous rather than categorical variables to avoid potential bias.

## 2. Methods

### 2.1. Patients

Patients who underwent gastric resection at a tertiary hospital between June 2006 and August 2017 were analyzed. The inclusion criteria were as follows: stage I–III GC, according to the American Joint Committee on Cancer staging system (8th edition),^[12]^ and negative resection margins. Exclusion criteria included malignancies within the past 5 years or concurrent malignancies, any anticancer treatment prior to surgery, severe infections within 4 weeks prior to surgery, and preexisting active autoimmune diseases.

This study was approved by the Institutional Review Board (IRB) of Kyung Hee University Hospital at Gangdong (IRB 2021-05-015). Given that this was a retrospective study, the requirement for informed consent was waived by the IRB.

### 2.2. Baseline clinical characteristics

Data on clinicopathological parameters, including age, sex, performance status, body mass index, tumor site, tumor size, nodal invasion, stage, type of gastrectomy, histological classification based on Lauren criteria,^[13]^ vascular invasion, lymphatic invasion, and perineural invasion (PNI), were analyzed. White blood cell count, hemoglobin level, platelet count, MPV level, and serum albumin level (ALB) tested within 7 days before surgery were analyzed.

### 2.3. Measurement of MPV

All blood samples for MPV measurement were collected, handled (e.g., tube filling and mixing), and processed in the same manner. Based on the local guidelines for laboratory testing, ethylenediaminetetraacetic acid-anticoagulated blood samples were processed at room temperature within 1 hour of venous sampling. An LH 1502 impedance counter (Beckman Coulter, Inc., Miami, FL) was used to measure MPV levels. A normal MPV range was established in our laboratory. Regular internal and external quality controls were also used.^[14,15]^

### 2.4. Statistical analysis

Clinicopathological parameters are expressed as medians, with interquartile ranges in parentheses. Correlations between MPV levels and clinicopathological parameters were determined using Pearson correlation coefficient. The Mann–Whitney U test or Kruskal–Wallis test was used for intergroup comparison. Multicollinearity in the covariates was determined by calculating the variance inflation factor (VIF).

Harrell concordance index (C-index) was used to assess the model’s discriminative capacity. Decision curve analyses (DCAs) were performed to verify the clinical utility of the models for the 5-year OS and DFS. For DCA, bootstrap analysis with 500 resamples was performed. Finally, nomograms were constructed using the established models to predict OS and DFS in patients with GC. The nomograms were internally validated using calibration curves. Statistical analyses were performed by a statistician (W.E.) among the authors. All statistical analyses were performed using R package (r-project.org).

## 3. Results

### 3.1. Patients’ clinical characteristics

**Figure 1.**
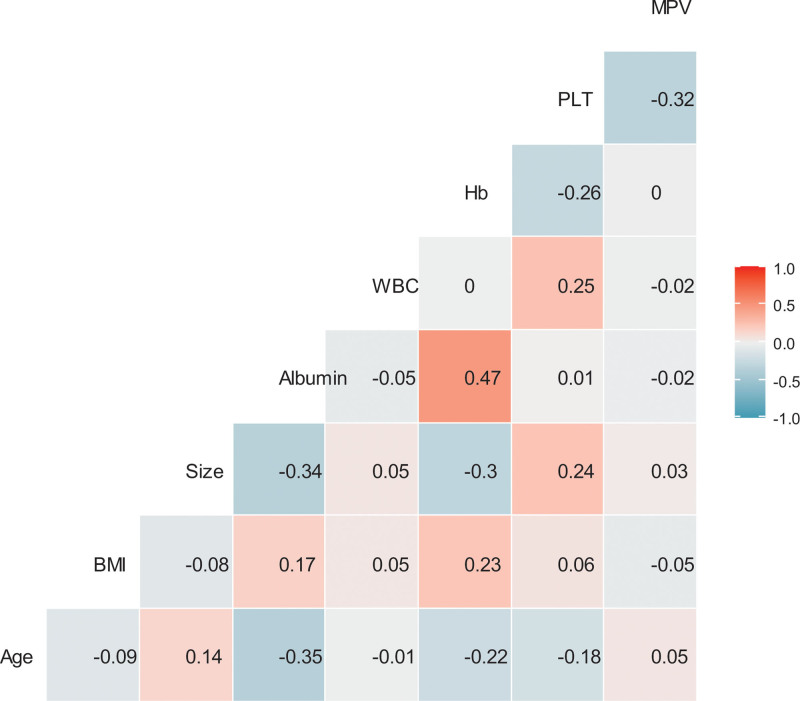
Correlation coefficients between mean platelet volume and clinical parameters. BMI = body mass index, Hb = hemoglobin concentration, MPV = mean platelet volume, PLT = platelet count, WBC = white blood cell count.

The clinicopathological parameters are shown in Table 1. The median age of the patients was 61 years. In total, 245 (61.1%) patients had stage I cancer, 74 (18.5%) had stage II cancer, and 82 (20.4%) had stage III cancer. Pearson correlation coefficient analysis showed no significant correlation between the MPV levels and clinicopathological parameters (Fig. 1). Similarly, using Mann–Whitney U tests or Kruskal–Wallis tests, no significant differences were found in MPV levels according to clinicopathological parameters. Regarding stage, the median MPV levels were 8.1 fL in stage I cancer, 7.8 fL in stage II cancer, and 7.8 fL in stage III cancer; thus, no significant difference was observed in the MPV level between stages by applying the Bonferroni method. Regarding lifestyle and underlying disorders, there were no significant differences in MPV according to smoking history, hypertension, diabetes mellitus, or obesity (Table 2).

**Table 1:** Patients’ characteristic.

Variables	Median (IQR): or n (%)
Age, yr	61 (52–70)
Sex	
Male	267 (66.6%)
Female	134 (33.4%)
Performance status	
0/1	319 (79.6%)
2/3	82 (20.4%)
Body mass index, kg/m^2^	23.7 (21.3–25.8)
Site of tumor	
Upper	41 (10.2%)
Middle	165 (41.1%)
Lower	189 (47.1%)
Diffuse	6 (1.5%)
Size of tumor, cm	3.0 (2.0–5.5)
Nodal invasion	
No	261 (65.1%)
Yes	140 (34.9%)
Stage	
I	245 (61.1%)
II	74 (18.5%)
III	82 (20.4%)
Gastrectomy	
Partial	316 (78.8%)
Total	85 (21.2%)
Histology	
Intestinal	197 (49.1%)
Diffuse	96 (23.9%)
Mixed	93 (23.2%)
Unknown	15 (3.7%)
Vascular invasion	
No	381 (95.0%)
Yes	20 (5.0%)
Lymphatic invasion	
No	268 (66.8%)
Yes	133 (33.2%)
Perineural invasion	
No	367 (91.5%)
Yes	34 (8.5%)
White blood cell, per μL	6500 (5400–7800)
Anemia*	
No	254 (63.3%)
Yes	147 (36.7%)
Platelet, ×10^3^/μL	235.0 (203.0–278.0)
Albumin, g/dL	4.1 (3.9–4.3)
Adjuvant chemotherapy	
No	256 (63.8%)
Yes	145 (36.2%)

IQR = interquartile range.

* Cutoff points were 12 g/dL in female patients and 13 g/dL in male patients.

**Table 2:** Mean platelet volume according to the clinicopathological parameters.

Variables	Mean platelet volume (fL)	
	Median (IQR)	*P* value
Age, yr		
<65	8.1 (7.5–11.9)	.877
≥65	8.0 (7.4–8.8)	
Sex		
Male	8.0 (7.4–8.8)	.352
Female	8.1 (7.5–9.0)	
Body mass index, kg/m^2^		
<18.5	8.0 (7.5–9.1)	.996
≥18.5	8.0 (7.5–8.8)	
Body mass index, kg/m^2^		
<30	8.0 (7.5–8.8)	.926
≥30	8.1 (7.4–8.6)	
Smoker		
Never	8.1 (7.5–8.8)	.309
Current/former	7.9 (7.5–8.8)	
Hypertension		
No	8.0 (7.5–8.7)	.988
Yes	8.0 (7.4–9.1)	
Diabetes mellitus		
No	8.0 (7.4–8.7)	.598
Yes	8.0 (7.5–9.3)	
Nodal invasion		
No	8.1 (7.5–8.9)	.175
Yes	7.9 (7.4–8.6)	
Stage		
I	8.1 (7.6–8.9)	.065
II	7.8 (7.5–8.8)	
III	7.8 (7.4–8.7)	
Histology		
Intestinal	7.9 (7.4–8.5)	.066
Others	8.1 (7.5–9.2)	
Vascular invasion		
No	8.0 (7.5–8.8)	.915
Yes	7.9 (7.7–8.7)	
Lymphatic invasion		
No	8.0 (7.4–8.8)	.466
Yes	8.1 (7.5–8.8)	
Perineural invasion		
No	8.0 (7.4–8.7)	.064
Yes	8.5 (7.6–9.4)	
Albumin, g/dL		
<3.5	8.0 (7.5–8.8)	.675
≥3.5	8.2 (7.2–9.6)	

IQR = interquartile range.

### 3.2. Cox regression of the risk factors of OS and DFS

The median follow-up time with interquartile range was 72.5 months (29.5–97.0 months). The significant variables in the univariate Cox regression analysis of OS were age, performance status, underweight, tumor size, nodal invasion, stage, vascular invasion, lymphatic invasion, PNI, anemia, ALB, and MPV levels (Table 3). Multivariate Cox regression revealed the following significant variables with respective hazard ratios (HRs) and 95% confidence intervals: age (1.05; 1.03–1.07; *P* < .001), stage (3.63; 2.30–5.73; *P* < .001), ALB (0.32; 0.21–0.48; *P <* .001), PNI (2.42; 1.31–4.49; *P <* .001), and MPV level (0.79; 0.62–0.99; *P* = .048). The VIFs for age, stage, ALB, PNI, and MPV were 1.07, 1.14, 1.09, 1.13, and 1.02, respectively (Table 4).

**Table 3:** Univariate Cox regression analyses of survival outcomes.

Variables	Overall survival		Disease-free survival	
	HR (95% CI)	*P* value	HR (95% CI)	*P* value
Age, yr *	1.06 (1.03–1.08)	<.001	1.05 (1.03–1.07)	<.001
Sex (female vs male)	0.74 (0.47–1.18)	.210	0.63 (0.40–1.00)	.050
Performance status (2/3 vs 0/1)	2.00 (1.25–3.22)	.004	1.80 (1.15–2.83)	.011
Underweight (yes vs no)†	1.98 (1.03–3.83)	.042	1.76 (0.92–3.40)	.089
Size of tumor, cm*	1.18 (1.13–1.23)	<.001	1.18 (1.13–1.23)	<.001
Nodal invasion (yes vs no)	3.50 (2.27–5.39)	<.001	3.98 (2.63–6.02)	<.001
Stage (III vs I/II)	5.56 (3.64–8.49)	<.001	5.95 (3.98–8.90)	<.001
Histology (intestinal vs others)	0.89 (0.59–1.36)	.604	0.91 (0.58–1.36)	.588
Vascular invasion (yes vs no)	3.39 (1.75–6.56)	<.001	4.07 (2.22–7.45)	<.001
Lymphatic invasion (yes vs no)	3.52 (2.30–5.41)	<.001	3.37 (2.32–5.20)	<.001
Perineural invasion (yes vs no)	2.72 (1.53–4.83)	.001	2.57 (1.48–4.47)	<.001
WBC count, per μL*	1.00 (0.99–1.00)	.737	1.00 (1.00–1.00)	.288
Anemia (yes vs no)‡	3.19 (2.08–4.90)	<.001	3.06 (2.04–4.59)	<.001
Albumin, g/dL*	0.21 (0.15–0.29)	<.001	0.17 (0.11–0.45)	<.001
Platelet count, ×10^3^/μL*	1.00 (0.99–1.00)	.286	1.00 (0.99–1.00)	.161
Mean platelet volume, fL*	0.77 (0.61–0.98)	.037	0.77 (0.62–0.96)	.021

CI = confidence interval, HR = hazard ratio, WBC = white blood cell.

* Continuous variable.

† Body mass index <18.5 kg/m^2^.

‡ Cutoff points are 12 g/dL in female patients and 13 g/dL in male patients.

**Table 4:** Multivariate Cox regression analyses of survival outcomes.

Variables	Overall survival		Disease-free survival	
	HR (95% CI)	*P* value	HR (95% CI)	*P* value
Age, yr*	1.05 (1.03–1.07)	<.001	1.04 (1.02–1.06)	<.001
Stage (III vs I/II)	3.63 (2.30–5.73)	<.001	4.11 (2.67–6.32)	<.001
Albumin, g/dL*	0.32 (0.21–0.48)	<.001	0.27 (0.17–0.41)	<.001
Perineural invasion (yes vs no)	2.42 (1.31–4.49)	.005	2.06 (1.14–3.72)	.017
Mean platelet volume, fL*	0.79 (0.62–0.99)	.048	0.75 (0.61–0.94)	.011

CI = confidence interval, HR = hazard ratio.

* Continuous variable.

Using univariate Cox regression analysis for DFS, the same variables that were significant in the OS analysis, except for underweight, were identified as significant (Table 3). Multivariate survival analysis using the Cox regression method revealed that age (1.04; 1.02–1.06; *P* < .001), stage (4.11; 2.67–6.32; *P* < .001), ALB (0.27; 0.17–0.41; *P* < .001), PNI (2.06; 1.14–3.72; *P* = .017), and MPV level (0.75; 0.61–0.94; *P* = .011) were significant determinants of DFS. The VIFs for age, stage, ALB, PNI, and MPV were 1.12, 1.11, 1.11, 1.13, and 1.02, respectively (Table 4).

### 3.3. Establishment and validation of prognostic models

The MPV model was established using 5 covariates (age, stage, ALB, PNI, and MPV level), and the baseline model was established using the same covariates as the MPV model, except for the MPV level.

**Figure 2.**
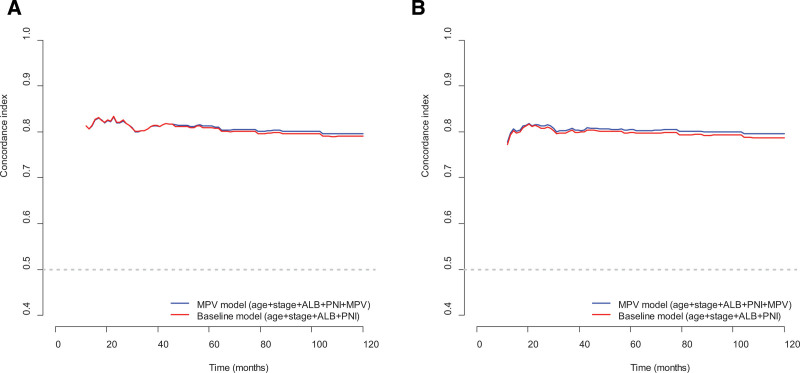
Harrell concordance indices of models over time. (A) Overall survival; (B) Disease-free survival. ALB = serum albumin level, MPV = mean platelet volume, PNI = perineural invasion.

The C-indices for OS were 0.82 in the MPV model and 0.81 in the baseline model. Similarly, the C-indices of the MPV model and the baseline model for DFS were 0.81 and 0.80, respectively. Thus, these findings suggest higher C-indices for OS and DFS in the MPV model than those in the baseline model. In addition, the C-index of the MPV model for determining OS and DFS was higher than that of the baseline model over a 10-year period (Fig. 2).

**Figure 3.**
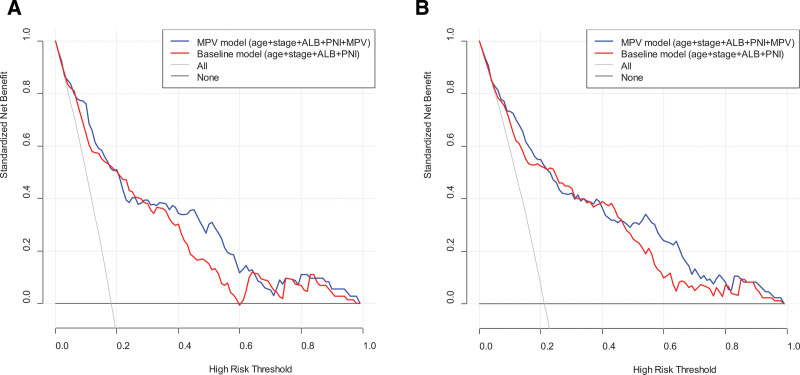
Decision curve analysis to calculate the clinical net benefit of the models for 5-yr survivals. (A) Overall survival; (B) Disease-free survival. ALB = serum albumin level, All = a line indicating that all patients survived, MPV = mean platelet volume, None = a line indicating that none of the patients survived, PNI = perineural invasion.

DCA was performed to verify the clinical utility of the models for the 5-year OS and DFS. We found that the MPV model had a greater net advantage than the baseline model at almost all threshold probabilities for both OS and DFS (Fig. 3).

**Figure 4.**
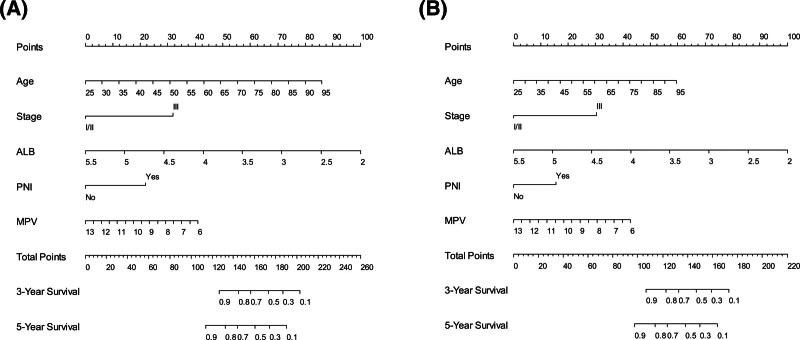
Nomograms predicting 3- and 5-yr survival. (A) Overall survival; (B) Disease-free survival. ALB = serum albumin level, MPV = mean platelet volume, PNI = perineural invasion.

**Figure 5.**
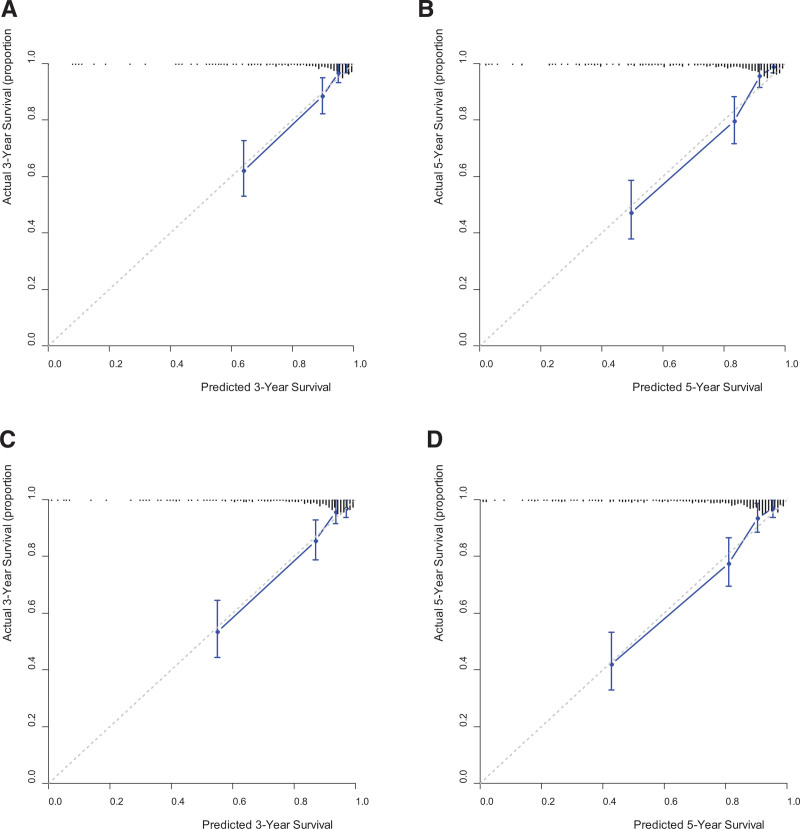
Calibration curves predicting survival. (A) 3-yr OS; (B) 5-yr OS; (C) 3-year DFS; (D) 5-year DFS. DFS = disease-free survival, OS = overall survival.

Finally, nomograms that could predict the survival outcomes were established using the MPV model (Fig. 4). When validating the nomogram using calibration curves, the predicted survival closely matched the actual survival (Fig. 5).

## 4. Discussion

This study aimed to evaluate the prognostic potential of the MPV in patients with stage I–III GC who underwent gastrectomy. In this study, MPV was found to be an important determinant of OS and DFS.

Platelets promote cancer progression through tumor angiogenesis, cancer cell proliferation, and resistance to apoptosis.^[3]^ The stimulatory effect of platelets on tumor progression was validated in a study conducted by Algra et al, which showed that regular use of aspirin, an antiplatelet agent, reduced the risk of distant metastasis.^[3,16]^ With regard to platelet size, the MPV level may have prognostic significance in certain tumor types; however, its prognostic significance in overall malignancy remains unclear.^[5]^

In GC, although the pretreatment MPV level is not a prognostic determinant for OS based on multivariate analysis,^[8–11]^ the clinical value of the MPV level remains uncertain given the limited number of available studies, various cutoff values, and various TNM stages. Similarly, in a recent meta-analysis performed by Chen et al, although they emphasized that the MPV level had a strong association with OS, it included studies with various cutoff values (i.e., 9.83 to 11.65 fL), adopted HRs derived from univariate Cox regression rather than from multivariate regression, and did not go through a validation process, which limited the ability to reach conclusions.^[5]^

In the present study, using multivariate Cox regression for OS, the HR for 1 unit change in the MPV level was 0.79 (*P* = .048) (Table 4). Therefore, the results of our study differ from those of previous studies.^[5,8–11]^ However, this study has the advantage of evaluating the clinical significance of MPV levels as a continuous variable rather than a categorical variable. It is well known that the optimal cutoff values obtained by minimizing the *P* value are prone to bias, thus limiting their application in different cohorts.^[5,17]^

Similarly, the HR per unit change in the MPV level was 0.75 (*P* = .011) using multivariate Cox regression for DFS (Table 4). Additionally, an association was observed between the decrease in MPV levels and the decrease in the probability of DFS in the established monogram (Fig. 4B). However, in Shen et al study, which included patients with surgically resectable stage I to IV GC, a high preoperative MPV level predicted poor DFS (HR 2.41, *P* = .001) by applying multivariate Cox regression analyses.^[8]^ Therefore, the results of the present study contradict those of a previous study conducted by Shen et al. However, considering the differences in the methods of treating MPV levels (continuous vs categorized variables) and stages (stages I–III vs stages I–IV), the results of the present study cannot be directly compared with those of previous studies.

Similar to the results of our study, the association between low MPV levels and poor survival outcomes has been reported in various types of solid tumors, including renal cell carcinoma, hepatoma, bladder cancer, lung cancer, oropharyngeal cancer, pancreatic cancer, colorectal cancer, and malignant lymphoma.^[18–22]^ However, the exact mechanism by which reduction in MPV levels is associated with poor survival outcomes remains unclear. The proposed hypotheses are as follows: first, platelet activation increases cancer-associated inflammation, leading to platelet accumulation with increased MPV levels at the site of inflammation, resulting in decreased MPV levels in the peripheral blood.^[23,24]^ Similarly, excessive production of proinflammatory cytokines affects megakaryopoiesis by producing platelets with reduced MPV levels.^[25]^ In a study conducted by Gasparyan et al showed that decreased MPV levels were associated with the severity of inflammatory diseases, and this effect could be reversed by anti-inflammatory therapy.^[26]^ These results indicated that a decrease in MPV reflects platelet activation and inflammation. Second, cytokines released from tumor cells may promote vascular endothelial thrombus formation, thereby increasing the consumption of platelets with increased MPV levels and eventually decreasing MPV levels.^[27]^ Finally, low MPV levels have been associated with an increased risk of thrombosis, which is one of the leading causes of death in cancer patients.^[22,28]^ However, further investigation is required to elucidate the association between MPV levels and GC prognosis.

In the present study, no significant correlation was observed between the MPV levels and clinicopathological parameters. With regard to the association between the MPV level and stage, the results of our study were consistent with those of previous studies that showed no significant differences in MPV levels among GC stages.^[29,30]^ Regarding the association between MPV levels and platelet counts, the results of the present study were compatible with those of previous studies, which showed no correlation between MPV levels and platelet count under pathological conditions.^[31,32]^ Regarding the association between MPV levels and underlying disorders, there were no significant differences in MPV levels according to hypertension, diabetes, or obesity status. The results of our study contradict those of previous studies, suggesting that MPV levels can be affected by hypertension, diabetes, impaired fasting glucose, and obesity.^[5,33–35]^ However, considering the relatively healthy patients without malignant tumors in previous studies, the results of the present study cannot be directly compared with those of previous studies.

In this study, in addition to MPV, age, stage, ALB, and PNI were independent prognostic factors for OS and DFS in the multivariate Cox regression analysis. The prognostic value of age, stage, ALB, and PNI as determinants of survival outcomes in patients with GC has been previously reported.^[36]^ In the present study, the C-indices for OS and DFS were higher in the MPV model than in the baseline model. By performing DCA to verify the clinical utility of the models for 5-year OS and DFS, we found that the MPV model had a greater net advantage than the baseline model at almost all the threshold probabilities for both OS and DFS. These results suggest that MPV levels have a clinical value in determining survival outcomes. Using the MPV model, nomograms were established to predict survival outcomes and were verified using calibration curves. Although age and ALB comprised most of the total scores in the nomograms, patients with an MPV level of 6 scored approximately 43, indicating the clinical value of MPV level as a survival predictor.

The strengths of this study are as follows. First, the MPV level, when treated as a continuous variable, was an important determinant of OS and DFS. To our knowledge, no significant association between MPV and OS or DFS has been reported in patients with stage I–III GC using multivariate Cox regression. Second, the C-index of the MPV model was higher than that of the baseline model for both OS and DFS, demonstrating the superior discriminative capacity of the model including MPV levels. When the nomogram was established using the MPV model, the calibration curve showed that the predicted survival was in close agreement with actual survival. Therefore, the results of the present study imply the clinical significance of MPV level as a predictor of survival outcomes in GC.

However, the present study has some limitations; therefore, caution should be exercised when interpreting the results. First, because this study was conducted retrospectively, data omission may have affected the results. Second, although potential bias was controlled, this was a single-center data analysis without external validation.

In conclusion, age, stage, ALB level, PNI, and MPV are independent prognostic factors for OS and DFS. The C-index for OS and DFS of the MPV model was higher than that of the baseline model, implying that the MPV level is a clinically significant predictor of survival in GC. However, because MPV levels have not been well-tested in GC, external validation prior to clinical application is a prerequisite.

## Author contributions

Conceptualization: S.A., W.E.; Data curation: S.A., W.E., G.Y.H., S.P.; Funding acquisition: Not available; Investigation: S.A., W.E., G.Y.H., S.P., S.L.; Methodology: S.A., S.L.; Supervision: W.E.; Writing – original draft: S.A.; Writing – review and editing: S.A., W.E., G.Y.H., S.P., S.L.

## References

[1] Balachandran VP Gonen M Smith JJ. Nomograms in oncology: more than meets the eye. Lancet Oncol. 2015;16:e173–80. 25846097 10.1016/S1470-2045(14)71116-7 PMC4465353

[2] Oñate-Ocaña LF Aiello-Crocifoglio V Gallardo-Rincón D. Serum albumin as a significant prognostic factor for patients with gastric carcinoma. Ann Surg Oncol. 2007;14:381–9. 17160496 10.1245/s10434-006-9093-x

[3] Sabrkhany S Kuijpers MJE Oude Egbrink MGA. Platelets as messengers of early-stage cancer. Cancer Metastasis Rev. 2021;40:563–73. 33634328 10.1007/s10555-021-09956-4 PMC8213673

[4] Ikeda M Furukawa H Imamura H. Poor prognosis associated with thrombocytosis in patients with gastric cancer. Ann Surg Oncol. 2002;9:287–91. 11923136 10.1007/BF02573067

[5] Chen X Li J Zhang X. Prognostic and clinicopathological significance of pretreatment mean platelet volume in cancer: a meta-analysis. BMJ Open. 2020;10:e037614. 10.1136/bmjopen-2020-037614 PMC7592286 33109647

[6] Giannakeas V Kotsopoulos J Brooks JD. Platelet count and survival after cancer. Cancers (Basel). 2022;14:549. 35158817 10.3390/cancers14030549 PMC8833779

[7] Park Y Schoene N Harris W. Mean platelet volume as an indicator of platelet activation: methodological issues. Platelets. 2002;13:301–6. 12189016 10.1080/095371002220148332

[8] Shen XM Xia YY Lian L. Mean platelet volume provides beneficial diagnostic and prognostic information for patients with resectable gastric cancer. Oncol Letters. 2016;12:2501–6. 10.3892/ol.2016.4913 PMC5038875 27703523

[9] Lian L Xia YY Zhou C. Mean platelet volume predicts chemotherapy response and prognosis in patients with unresectable gastric cancer. Oncol Letters. 2015;10:3419–24. 10.3892/ol.2015.3784 PMC4665329 26788144

[10] Zhou X Xu L Huang Z. The hematologic markers as prognostic factors in patients with resectable gastric cancer. Cancer Biomark. 2016;17:359–67. 27434296 10.3233/CBM-160648 PMC13020502

[11] Song S Cong X Li F. The fibrinogen to mean platelet volume ratio can predict overall survival of patients with non-metastatic gastric cancer. J Gastr Cancer. 2018;18:368–78. 10.5230/jgc.2018.18.e36 PMC6310767 30607300

[12] Amin MB Greene FL Edge SB. The Eighth Edition AJCC cancer staging manual: continuing to build a bridge from a population-based to a more “personalized” approach to cancer staging. CA. 2017;67:93–9. 28094848 10.3322/caac.21388

[13] Lauren P. The two histological main types of gastric carcinoma: diffuse and so-called intestinal-type carcinoma. An attempt at a histo-clinical classification. Acta Pathologica et Microbiologica Scandinavica. 1965;64:31–49. 14320675 10.1111/apm.1965.64.1.31

[14] Harrison P Goodall AH. Studies on mean platelet volume (MPV) - new editorial policy. Platelets. 2016;27:605–6. 27612027 10.1080/09537104.2016.1225467

[15] Noris P Melazzini F Balduini CL. New roles for mean platelet volume measurement in the clinical practice? Platelets. 2016;27:607–12. 27686008 10.1080/09537104.2016.1224828

[16] Algra AM Rothwell PM. Effects of regular aspirin on long-term cancer incidence and metastasis: a systematic comparison of evidence from observational studies versus randomised trials. Lancet Oncol. 2012;13:518–27. 22440112 10.1016/S1470-2045(12)70112-2

[17] Hacker UT Hasenclever D Linder N. Prognostic role of body composition parameters in gastric/gastroesophageal junction cancer patients from the EXPAND trial. J Cachexia Sarcopenia Mus. 2020;11:135–44. 10.1002/jcsm.12484 PMC7015239 31464089

[18] Yagyu T Saito H Sakamoto T. Decreased mean platelet volume predicts poor prognosis in patients with pancreatic cancer. BMC Surg. 2021;21:8. 33407353 10.1186/s12893-020-00976-5 PMC7788764

[19] Delago D Knittelfelder O Jakse G. The decreased mean platelet volume is associated with poor prognosis in patients with oropharyngeal cancer treated with radiotherapy. Radiat Oncol. 2020;15:259. 33160368 10.1186/s13014-020-01702-4 PMC7648964

[20] Chang J Lin G Ye M. Decreased mean platelet volume predicts poor prognosis in metastatic colorectal cancer patients treated with first-line chemotherapy: results from mCRC biomarker study. BMC Cancer. 2019;19:15. 30612568 10.1186/s12885-018-5252-2 PMC6322328

[21] Zhou S Ma Y Shi Y. Mean platelet volume predicts prognosis in patients with diffuse large B-cell lymphoma. Hematol Oncol. 2018;36:104–9. 28736928 10.1002/hon.2467

[22] Riedl J Kaider A Reitter EM. Association of mean platelet volume with risk of venous thromboembolism and mortality in patients with cancer. Results from the Vienna Cancer and Thrombosis Study (CATS). Thromb Haemost. 2014;111:670–8. 24306221 10.1160/TH13-07-0603

[23] Grivennikov SI Greten FR Karin M. Immunity, inflammation, and cancer. Cell. 2010;140:883–99. 20303878 10.1016/j.cell.2010.01.025 PMC2866629

[24] Gasparyan AY Sandoo A Stavropoulos-Kalinoglou A. Mean platelet volume in patients with rheumatoid arthritis: the effect of anti-TNF-α therapy. Rheumatol Int. 2010;30:1125–9. 20066426 10.1007/s00296-009-1345-1

[25] Refaai MA Phipps RP Spinelli SL. Platelet transfusions: impact on hemostasis, thrombosis, inflammation and clinical outcomes. Thromb Res. 2011;127:287–91. 21093892 10.1016/j.thromres.2010.10.012 PMC3062691

[26] Gasparyan AY Ayvazyan L Mikhailidis DP. Mean platelet volume: a link between thrombosis and inflammation? Curr Pharm Des. 2011;17:47–58. 21247392 10.2174/138161211795049804

[27] Falanga A Panova-Noeva M Russo L. Procoagulant mechanisms in tumour cells. Best Pract Res Clin Haematol. 2009;22:49–60. 19285272 10.1016/j.beha.2008.12.009

[28] Palacios-Acedo AL Langiu M Crescence L. Platelet and cancer-cell interactions modulate cancer-associated thrombosis risk in different cancer types. Cancers (Basel). 2022;14:730. 35159000 10.3390/cancers14030730 PMC8833365

[29] Pietrzyk L Plewa Z Denisow-Pietrzyk M. Diagnostic power of blood parameters as screening markers in gastric cancer patients. Asian Pac J Cancer Prev. 2016;17:4433–7. 27797257

[30] Kilinçalp S Ekiz F Başar O. Mean platelet volume could be possible biomarker in early diagnosis and monitoring of gastric cancer. Platelets. 2014;25:592–4. 23537073 10.3109/09537104.2013.783689

[31] Thompson CB Jakubowski JA. The pathophysiology and clinical relevance of platelet heterogeneity. Blood. 1988;72:1–8. 3291975

[32] Korniluk A Koper-Lenkiewicz OM Kamińska J. Mean platelet volume (MPV): new perspectives for an old marker in the course and prognosis of inflammatory conditions. Mediators Inflamm. 2019;2019:9213074. 31148950 10.1155/2019/9213074 PMC6501263

[33] Coban E Ozdogan M Yazicioglu G. The mean platelet volume in patients with obesity. Int J Clin Pract. 2005;59:981–2. 16033624 10.1111/j.1742-1241.2005.00500.x

[34] Varol E Akcay S Icli A. Mean platelet volume in patients with prehypertension and hypertension. Clin Hemorheol Microcirc. 2010;45:67–72. 20571231 10.3233/CH-2010-1327

[35] Zuberi BF Akhtar N Afsar S. Comparison of mean platelet volume in patients with diabetes mellitus, impaired fasting glucose and non-diabetic subjects. Singapore Med J. 2008;49:114–6. 18301837

[36] An S Eo W Kim YJ. Muscle-related parameters as determinants of survival in patients with stage I-III gastric cancer undergoing gastrectomy. J Cancer. 2021;12:5664–73. 34405026 10.7150/jca.61199 PMC8364646

